# Humanization of Drug Metabolism in the *Plasmodium berghei* Mouse Model for Antimalarial Drug Discovery

**DOI:** 10.1021/acsinfecdis.5c00681

**Published:** 2025-12-01

**Authors:** A. Kenneth MacLeod, Cristina Merino, Sara Viera-Morilla, Laura Frame, Amy Tavendale, Christina Duncan, Liam Ferguson, Erika G. Pinto, Frederick R. C. Simeons, Vanessa Gomez-Jimenez, Ana Belén García, Jennifer Riley, Yoko Shishikura, María Santos Martínez- Martínez, C. Roland Wolf, Kevin D. Read

**Affiliations:** † Drug Discovery Unit, Wellcome Centre for Anti-Infectives Research, School of Life Sciences, 3042 University of Dundee, Dundee DD1 5EH, U.K.; ‡ Global Health Medicines R&D, GSK, Tres Cantos, 28760 Madrid, Spain; § Division of Systems Medicine, Jacqui Wood Cancer Centre, School of Medicine, 3042 University of Dundee, Ninewells Hospital, Dundee DD2 4GD, U.K.

**Keywords:** malaria, drug discovery, drug metabolism, animal models, Plasmodium berghei

## Abstract

Resistance to artemisinin-based combination therapies (ACTs) is steadily increasing in malaria-endemic countries, and new medicines to treat this disease are urgently needed. Drug discovery efforts are hindered by species differences in drug metabolism as new chemical entities must survive metabolism by diverse enzymes across multiple species, enabling cures in preclinical disease models before progression to the clinic. Here, we show how the use of a mouse line extensively genetically humanized for enzymes of the cytochrome P450 superfamily and their transcriptional regulators, the “8HUM” line, can circumvent this issue and improve the translational accuracy of data generated. Engraftment of human erythrocytes into 8HUM/Rag2^–/–^, an immunocompromised version of the 8HUM line lacking mature T and B cells, was insufficient to permit infection with *Plasmodium falciparum*, and depletion of natural killer cells by antibody treatment did not alter this outcome. However, infection of 8HUM with *Plasmodium berghei* permitted assessment of drug efficacy against this *Plasmodium* species. Approved antimalarials were generally more metabolically stable in 8HUM than in wild-type mice. Major species differences between humans and mice in routes of metabolic elimination for quinine derivatives were removed with 8HUM. Therefore, the 8HUM *P. berghei* model described here will be of value early in the critical path for antimalarial drug discovery, improving alignment of drug metabolism with the clinical situation while bypassing mouse-specific issues of metabolism to facilitate proof-of-concept in vivo demonstration of efficacy, a key requirement for validation of new drug targets and chemical series.

## Graphical abstract

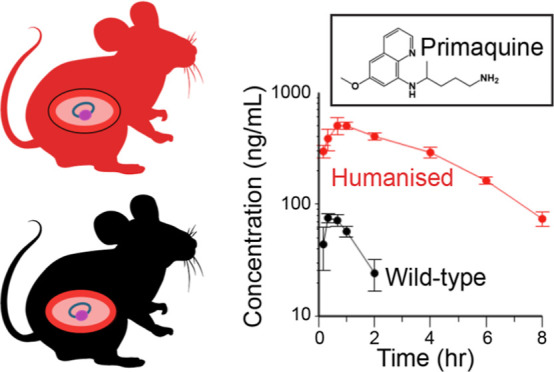

## Introduction

Malaria is a devastating disease caused by single-celled parasites of the *Plasmodium* genus, which primarily infect humans through bites of infected *Anopheles* mosquitoes. There were an estimated 263 million cases and 597,000 deaths globally in 2023, many of which involved children under the age of five.^1^ Five species of *Plasmodium* commonly infect humans, *Plasmodium falciparum*, *Plasmodium vivax,* *Plasmodium malariae*, *Plasmodium ovale,* and *Plasmodium knowlesi*, with the first of these by far the deadliest. Artemether combination therapy (ACT), where artemether or a derivative thereof is combined with one or two additional antimalarials, is the current first-line treatment recommended by the World Health Organization for uncomplicated falciparum malaria in all endemic countries. But since the first reports of its emergence in Western Cambodia,^2^ artemisinin resistance has spread widely, from this and multiple independent origins.^3,4^ New drugs to treat malaria are urgently needed.^5^

Due to an appropriate balance of physiological relevance, practicality, and cost, the mouse is the most widely used preclinical model for human antimalarial drug discovery, and the critical path usually includes demonstration of efficacy in a murine model of disease. *Plasmodium* typically has a narrow host range: species that cause disease in humans are quickly cleared by the immune system in mice.^6^ As a result, antimalarial drug discovery often involves infection of mice with alternative *Plasmodium* species such as *Plasmodium berghei*, *Plasmodium yoelii*, *Plasmodium chabaudi,* and *Plasmodium vinckei*. Preclinical in vivo efficacy assessment against the species that cause malaria in humans is possible but requires the use of nonhuman primates or humanized mouse models, the latter generated through intravenous or intraperitoneal injection of human tissues to permit infection.^6^ It was first demonstrated in 1995 that severe combined immunodeficient (SCID) mice, lacking mature T and B lymphocytes, could be engrafted with human erythrocytes (hE) and infected with *P. falciparum*, recreating human blood-stage disease, although rates of engraftment and parasitemia were low despite further immunomodulation through coadministration of human serum, inhibition of the macrophage response, or crossing with nonobese diabetic (NOD) mice defective in several aspects of immunity.^7–9^ Since this initial work, a variety of refinements have been described,^10^ including deletion of the interleukin 2 receptor gamma chain gene, *il2rg*, on an NOD/SCID background.^11^ This model, known commonly as NOD/SCID/gamma or NSG, shows a defective natural killer (NK) cell response and is now widely used in antimalarial drug discovery due to the observed high rates of hE engraftment and *P. falciparum* infection.^6,12^

We have previously described the 8HUM mouse line, which is extensively humanized for genes of the cytochrome P450 (CYP) superfamily and, as a result, metabolizes drugs much more like humans. In this line, genes encoding 33 mouse CYPs from the *Cyp1a*, 2c, 2d, and 3a families, together with the transcription factors pregnane x receptor (Pxr) and constitutive androstane receptor (Car), have been deleted and replaced with human *CYP1A1, CYP1A2, CYP2C9, CYP2D6, CYP3A4, CYP3A7*, PXR, and CAR.^13^ These CYP enzymes were selected because they are responsible for the vast majority of phase I metabolism of clinical significance in humans.^14–16^ The 8HUM model can be used to circumvent common drug development problems associated with rapid mouse-specific routes of metabolic elimination and improves the translation of results in relation to active metabolites and drug–drug interactions.^17^ This advance has the potential to significantly reduce the number of animals used in the early and preclinical stages of drug development while simultaneously improving the clinical relevance of data and increasing the chances of translational success. Recently, we have introduced an immunocompromised version of this model, 8HUM/Rag2^–/–^.^18^ Knockout of the Rag2 gene prevents maturation of T and B lymphocytes, allowing subcutaneous xenograft of human cell lines for assessment of anticancer drug activity. Here, we report the outcome of studies evaluating the engraftment of 8HUM/Rag2^–/–^ with hE to facilitate *P. falciparum* infection, and of infection of immunocompetent 8HUM with *P. berghei*, for the support of antimalarial drug discovery. In addition, we characterize the metabolism of selected antimalarial standards of care in 8HUM, demonstrating improved alignment with human data for these compounds.

## Results

### Immunodepletion by Rag2 Knockout Is Insufficient to Permit Human Erythrocyte Engraftment and Infection with *P. falciparum* in 8HUM

To determine whether 8HUM/Rag2^–/–^ could be engrafted with hE and subsequently infected with *P. falciparum*, we tested a protocol used routinely at GSK during evaluation of the efficacy of New Chemical Entities (NCEs).^12^ In a typical study, administration of hE to NSG mice once daily by intraperitoneal (i.p.) injection leads to displacement of approximately 50% of murine erythrocytes by approximately 10 days, allowing infection with *P. falciparum*. After a further 3 days, parasitemia levels of approximately 0.5% allow NCE efficacy to be tested. With 8HUM/Rag2^–/–^, however, hE engraftment levels were low (Figure 1A). On day 3, average engraftment in 8HUM/Rag2^–/–^ mice was around 20% and complete rejection generally occurred around 6–8 days after initiation, with half of the mice showing near-zero levels of engraftment by this stage. On day 17, only 6 of 25 mice showed >20% repopulation with human cells. This was in contrast to the control group of NSG mice, where robust engraftment was observed (Figure 1B). The six 8HUM/Rag2^–/–^, which showed some degree of engraftment, were infected with *P. falciparum* but no detectable parasitemia was observed over the following 7 days (Figure 1C), in contrast to the subset of NSG mice, which were infected as a positive control group (Figure 1D).

**Figure 1:**
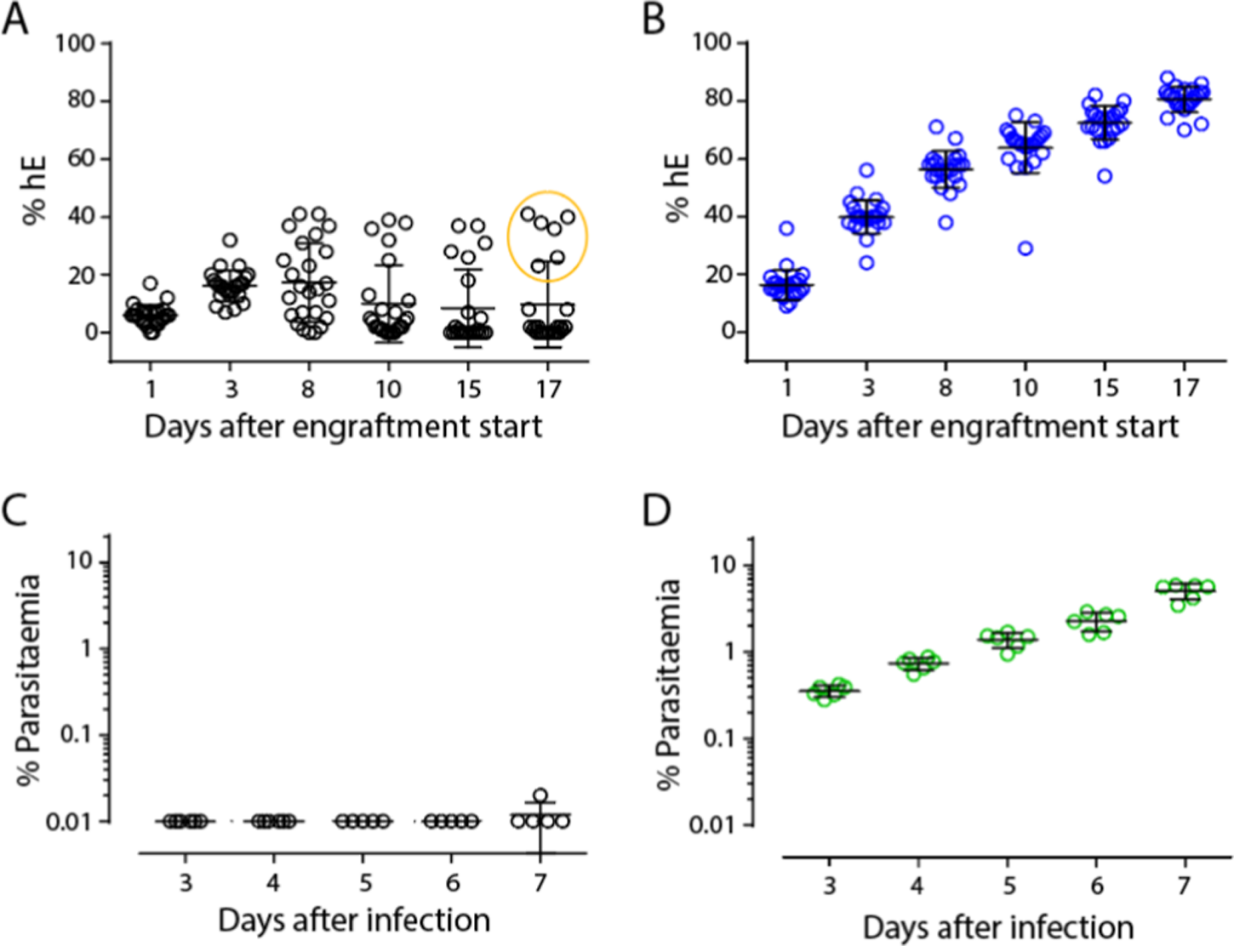
Immunodepletion by Rag2 knockout is insufficient to permit human erythrocyte engraftment and infection with *P. falciparum* in 8HUM. (A) Rate of engraftment of hE in 8HUM/Rag2^–/–^mice, *n* = 25. Individuals circled were those taken forward for *P. falciparum* infection. (B) Rate of engraftment of hE in NSG mice, *n* = 25. (C) Level of parasitemia in 8HUM/Rag2^–/–^mice after infection with *P. falciparum*, *n* = 6. (D) Level of parasitemia in NSG mice after infection with *P. falciparum*, *n* = 6. On all plots, circles show individual data, with lines showing mean ± standard deviation.

### Further Immunodepletion of 8HUM/Rag2^–/–^ by Antibody-Mediated Depletion of Natural Killer Cells Is Insufficient to Permit Human Erythrocyte Engraftment and Infection with *P. falciparum*

Based on the observation that, as in the NSG mouse model, complete elimination of natural killer (NK) cells on an NOD/SCID background through genetic deletion of *il2rg* improves hE engraftment rate,^11^ we tested whether depletion of these cells in 8HUM/Rag2^–/–^ using an NK-specific antibody had a similar effect. To determine whether and to what extent NK cells were depleted following antibody administration, we administered two doses of 600 μg of an anti-NK1.1 antibody to 8HUM/Rag2^–/–^ at 72 and 24 h before tissue collection. Based on published studies using the same anti-NK antibody,^19–21^ this regimen was expected to give maximal response. Following analysis of the spleen and whole blood, we observed that NK cells were substantially depleted in both tissues, in comparison to mice administered the IgG2a isotype control antibody (Figure 2A). To test whether this level of depletion was sufficient to permit hE engraftment, we continued with this dose level in a further experiment. With a view to titrating the amount of reagent required down in future studies, we also included a second group at a lower dose of 200 μg. Depletion of NK cells was substantial, although, perhaps due to the presence of hE and consequent activation of an immune response, observed levels were somewhat different from the previous experiment (Figure 2B).

**Figure 2:**
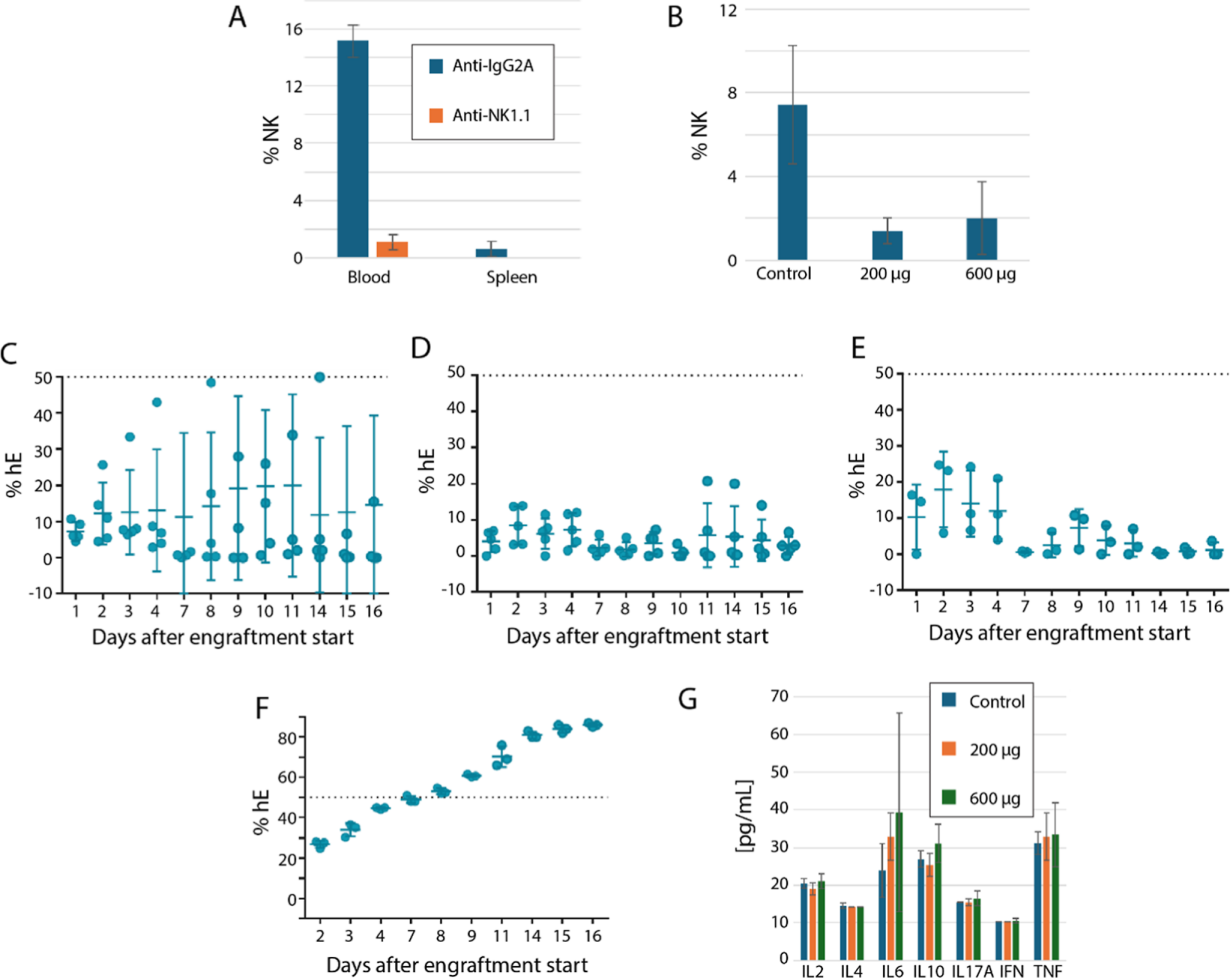
NK cell depletion insufficient to permit human erythrocyte engraftment and infection with *P. falciparum* in 8HUM/Rag2^–/–^. (A) NK cell levels in spleen and blood of 8HUM/Rag2^–/–^ following administration of two 600 μg doses of anti-NK1.1 antibody, *n* = 3 in each group. (B) NK cell levels in blood of 8HUM/Rag2^–/–^ administered NK cell blocking antibody, *n* = 3, in each group. Rates of human erythrocyte engraftment were determined for 8HUM/Rag2^–/–^ during treatment with 200, *n* = 5 (C) or 600, *n* = 5 (D) μg/dose anti-NK antibody, and in 8HUM/Rag2^–/–^, *n* = 3 (E) and NSG, *n* = 3 (F) without blocking antibody. (G) Cytokine profile for 8HUM groups. All plots show mean ± standard deviation.

Although engraftment to a level of >50% hE was observed in a single mouse at the lower antibody dose level, engraftment of all other 8HUM/Rag2^–/–^ was unsuccessful, whether at the 200 μg dose level (Figure 2C), 600 μg dose level (Figure 2D), or in the absence of antibody (Figure 2E). Engraftment rate in NSG mice without antibody treatment was consistent with previous experiments (Figure 2F). The cytokine profile was assessed in 8HUM/Rag2^–/–^ groups at the end of the experiment, after eight doses of antibody and 16 days of engraftment; however, the profile of all cytokines was similar in all three groups (Figure 2G). One data point of 1570 pg/mL for IL-6, from a mouse in the low-dose antibody group (not the outlier with >50% hE engraftment), was excluded from the plot shown in this figure.

### Short Course *P. berghei* Infection Allows Compound Efficacy Evaluation in 8HUM

As an alternative to the *P. falciparum* model, we assessed whether immunocompetent 8HUM could be infected with *P. berghei* in an experimental format that would facilitate compound evaluation against this *Plasmodium* species. This would therefore still allow, in early antimalarial drug discovery, a route to proof-of-concept for compound series, which suffer from mouse-specific metabolism issues, bypassing the need for medicinal chemistry optimization against mouse metabolism. We first tested a standard format, as widely used in CD-1 mice.^6,22^ Inoculums of 0.5–1.5 × 10^6^ infected erythrocytes resulted in similar levels of parasitemia after 3 days (∼2%) and 4 days (∼5%), which were also similar to those observed in CD-1. However, neurological effects associated with cerebral malaria were observed in the 8HUM, necessitating early termination of the study. To identify a window in which compound efficacy could be assessed, we evaluated a lower inoculum of 0.3 × 10^6^ infected erythrocytes (Figure 3A). At this lower inoculum, while parasitemia in untreated 8HUM reached approximately 10% after 5 days, a single dose of piperaquine 24 h after infection suppressed parasitemia to almost undetectable levels (Figure 3B).

**Figure 3:**
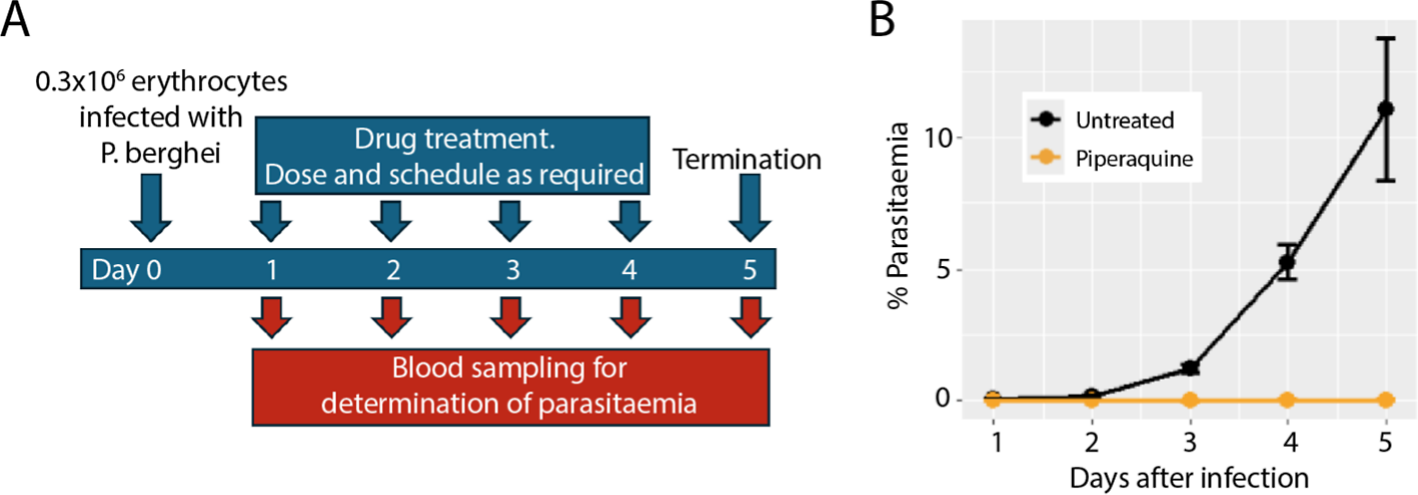
Modification of *P. berghei* model to a short course of infection allows compound efficacy evaluation in 8HUM. (A) Experimental design for short-course infection format. (B) Parasitemia in 8HUM infected with 0.3 × 10^6^ *P. berghei* and effect of treatment with a single dose of piperaquine on day 1, in comparison to the vehicle control group. The plot shows mean ± standard deviation for groups of *n*= 3 mice.

### In Vitro Metabolism of Approved Antimalarials in 8HUM

To characterize the metabolism of malaria standards of care in the 8HUM model, in vitro intrinsic clearance (CL_int_) of a panel of antimalarial drugs was determined using hepatic microsomal preparations from human, 8HUM, and WT mice. As expected for a set of approved medicines, many were highly metabolically stable with turnover below the limit of quantification (Figure 4A). Very little metabolism was observed for doxycycline, pyrimethamine, atovaquone, proguanil, and mefloquine. In contrast, three compounds were rapidly metabolized by human liver microsomes: amodiaquine, artemether, and artesunate. In the case of amodiaquine, which was much more stable in 8HUM than in human, with WT mouse CL_int_ falling between these values, metabolism to the active metabolite -desethylamodiaquine is known to occur rapidly in patients,^23^ and this biotransformation is mediated predominantly by CYP2C8.^24^ Seven metabolites of this compound were observed in incubation samples (Figure 4B), of which -desethylamodiaquine (M1) was by far the most abundant in samples from human and WT mouse incubations; however, this metabolite was present at very low levels in samples from incubations with 8HUM microsomes. This suggested that the higher CL_int_ observed with human and WT mice was due to -desethylation by CYP2C8 and unspecified murine Cyp/s, respectively. Low CL_int_ with 8HUM microsomes was consistent with the fact that neither CYP2C8 nor the majority of xenobiotic-metabolizing murine Cyps are present in this mouse line.

**Figure 4:**
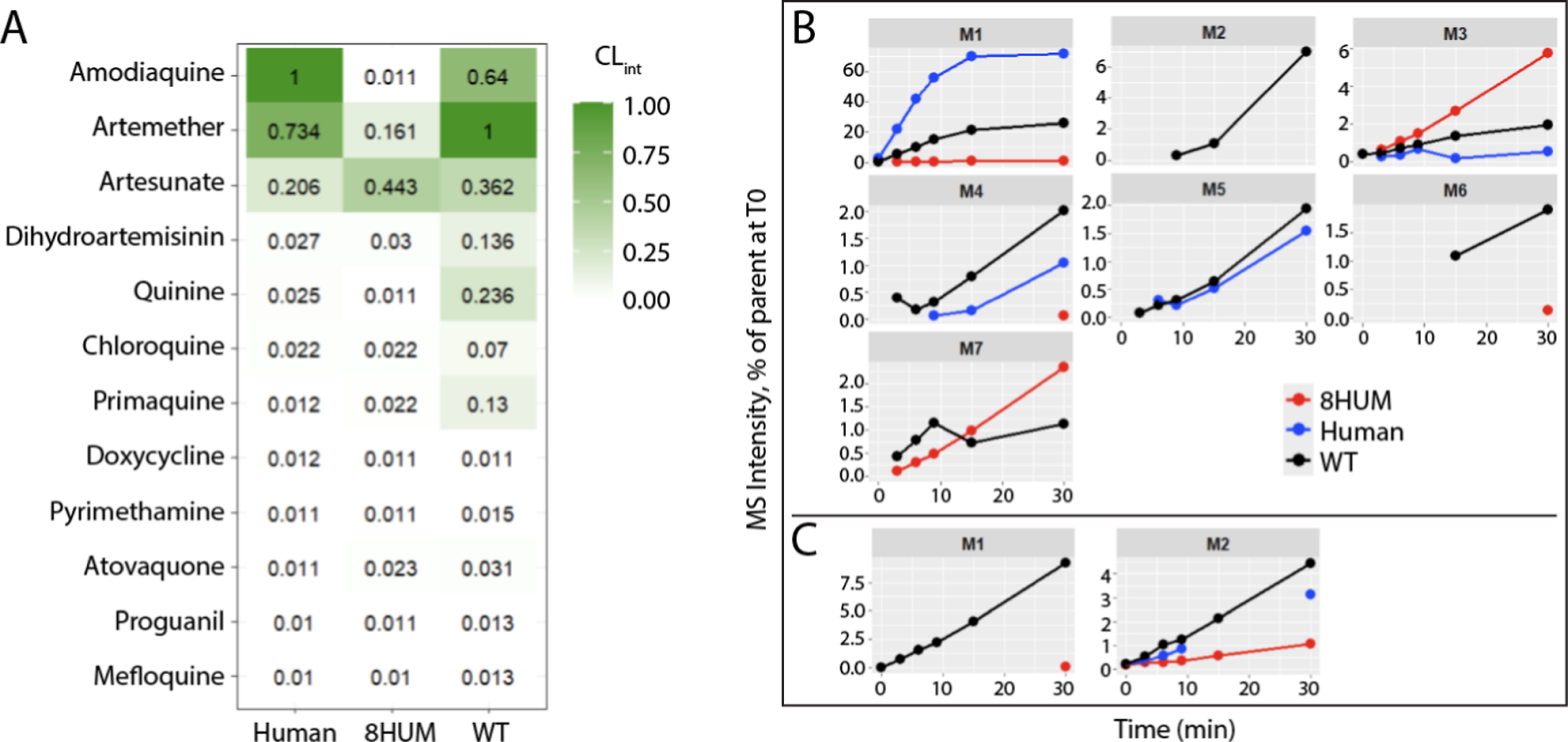
In vitro metabolism of approved antimalarials in 8HUM. (A) Intrinsic clearance with hepatic microsomes (mL/min/mg microsomal protein). (B) Metabolite profile of amodiaquine with hepatic microsomes. M1: de-ethylation (-C2H4), M2:2× de-ethylation (−C4H8) and oxidation (+O), M3: *N*-dealkylation (−C4H11N), M4: de-ethylation (−C2H4) and oxidation (+O), M5:2x de-ethylation (−C4H8), M6: oxidation (+O), M7: oxidation (+O). (C) Metabolite profile of chloroquine with hepatic microsomes. M1: oxidation (+O), M2: de-ethylation (−C2H4).

Four drugs were less stable with WT microsomes than with either human or 8HUM, three of which were quinolines: quinine, chloroquine, and primaquine. To further investigate this, we profiled the metabolites of chloroquine. This compound is known to be metabolized by CYP2C8 and CYP3A4 to -desethylchloroquine,^25,26^ a metabolite that, in our study, was observed in all three groups (Figure 4C, M2). Levels were lower with 8HUM microsomes than with human microsomes, consistent with the absence of CYP2C8 and lower abundance of CYP3A4 in 8HUM.^13^ However, more striking was the presence of an oxidized metabolite (M1), in relatively large quantities, in samples from WT mouse microsomes. This oxidation was localized to the quinoline group and suggested a species-specific route of metabolic elimination underlying the difference in CL_int_.

### Pharmacokinetics of Approved Antimalarials in 8HUM

We further investigated the species differences in quinoline drugs in vivo. Following an oral dose, and consistent with previous studies using a variety of anti-infective drugs,^17^ AUC_inf_ of chloroquine was higher (2.8-fold) in 8HUM than in WT mice (Figure 5 and Table 1). Five metabolites were detected, including -desethylchloroquine (M2) and -bidesethylchloroquine (M3). This pathway appeared to be the major route of metabolic elimination in 8HUM, as it is known to be in humans.^27^ An oxidized metabolite (M1) and a metabolite with both oxidation and -desethylation (M4) were observed in samples from both 8HUM and WT but at much higher levels in the latter. Consistent with the in vitro data (Figure 4C), this suggested that the rates of oxidation of the quinoline group were higher in WT mice.

**Figure 5:**
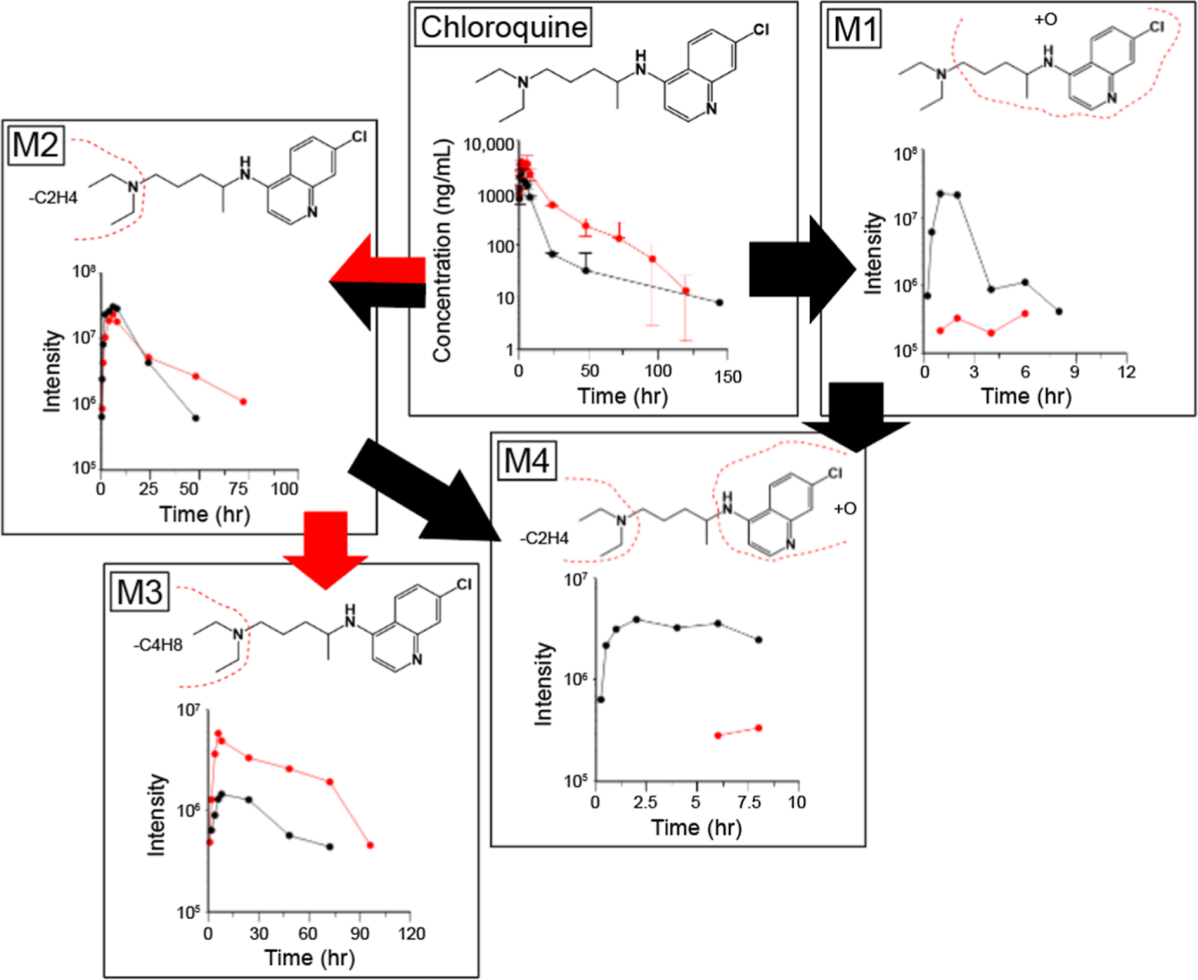
Pharmacokinetics of chloroquine and metabolites in 8HUM and WT mice. Chloroquine was administered PO at 5 mg/kg to 8HUM (red) and WT (black) mice (each *n* = 3) and serial blood samples taken for analysis. For parent drug, data shown are mean ± standard deviation of three replicates following quantitative analysis. For metabolites, samples were pooled across time points prior to LCMS data acquisition and values shown are MS peak intensity. Designation of M1 and M2 is consistent with the labeling used in Figure 4C. Arrows show preferential routes of metabolism for WT (black) and 8HUM (red).

**Table 1:** Pharmacokinetic Parameters in Whole Blood of Antimalarial Compounds Following Oral Administration to WT and 8HUM Mice^a^

compound	genotype	AUC_last_ (h*ng/mL)	*C*_max_ (ng/mL)	*T*_max_ (h)	B/P
chloroquine	WT	21,533 ± 1047	2526 ± 331	2.0 ± 0.0	3.03 ± 0.38
	8HUM	60,039 ± 3993	4465 ± 824	3.0 ± 2.6	3.88 ± 1.64
mefloquine	WT	35,370 ± 588	710 ± 64	4.7 ± 1.2	1.44 ± 0.07
	8HUM	99,166 ± 14,092	929 ± 110	7.3 ± 1.2	1.41 ± 0.16
primaquine	WT	101 ± 2	79.0 ± 5.0	0.6 ± 0.2	1.05 ± 0.19
	8HUM	2238 ± 46	534 ± 37	0.8 ± 0.2	1.01 ± 0.03
quinine	WT	569 ± 615	603 ± 506	0.3 ± 0.1	2.10 ± 0.12
	8HUM	15,390 ± 1496	1485 ± 639	10.7 ± 11.7	0.88 ± 0.05

a Parameters were calculated by noncompartmental analysis, whereas the blood-to-plasma ratio (B/P) was calculated on study by comparison of blood and plasma samples taken from the same animals at identical timepoints, as described in materials and methods.

At the administered oral dose of 5 mg/kg, AUC_inf_ of the 8-aminoquinoline, primaquine, was much higher (22-fold) in 8HUM than in WT mice (Figure 6 and Table 1). We observed two metabolites in blood samples from 8HUM and WT mice. Consistent with clinical observations, a metabolite conforming to carboxy-primaquine (M1) was the most abundant. The second metabolite (M2) contained hydroxylation in the aminoquinoline group and was much more abundant in 8HUM than in WT mice.

**Figure 6:**
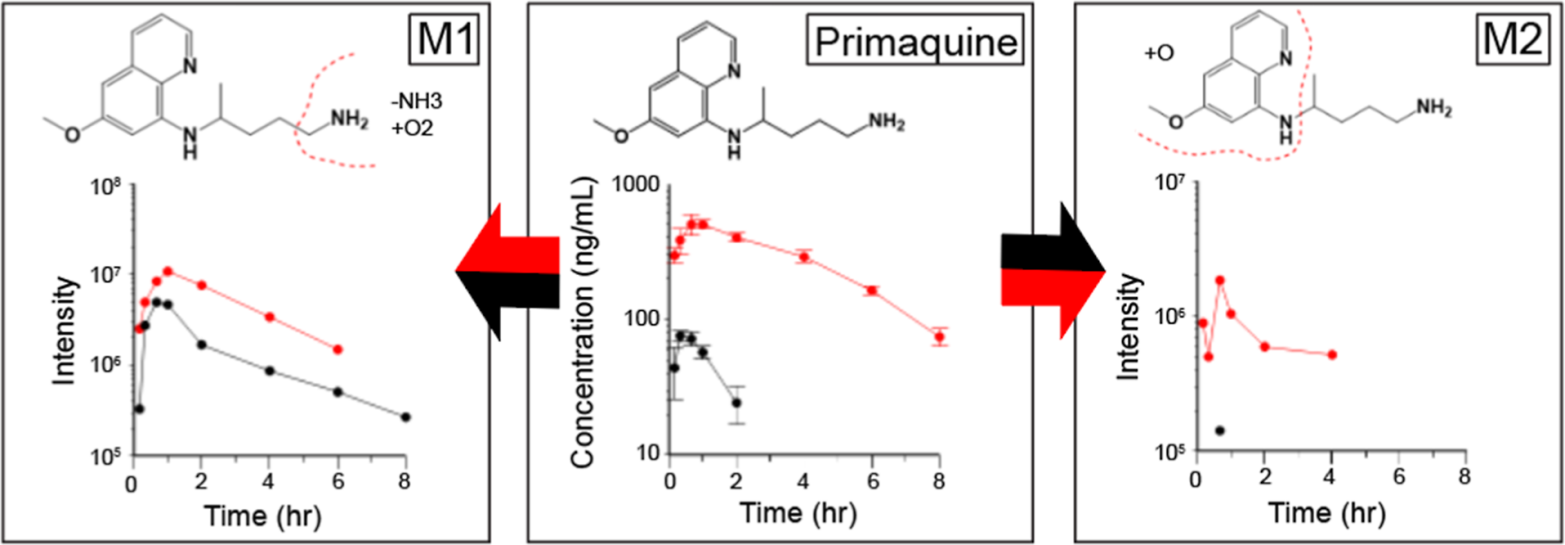
Pharmacokinetics of primaquine and metabolites in 8HUM and WT mice. Primaquine was administered PO at 5 mg/kg to 8HUM (red) and WT (black) mice (each *n* = 3) and serial blood samples taken for analysis. For parent drug, data shown are mean ± standard deviation of three replicates following quantitative analysis. For metabolites, samples were pooled across time points prior to LCMS data acquisition and values shown are MS peak intensity. Arrows show preferential routes of metabolism for WT (black) and 8HUM (red).

As for primaquine, the quinine AUC_inf_ was much higher (27-fold) in 8HUM than in WT mice (Figure 7 and Table 1). Four metabolites were observed in blood, although M1 (+H2) was likely not a metabolite but a common contaminant, dihydroquinine, which was specified present at “≤5%” in the Certificate of Analysis of the batch of quinidine administered. Two oxidations of the quinuclidine system were observed, M2 and M4, at higher abundance in 8HUM and WT, respectively. It was not possible to ascertain the exact identity of these metabolites with the spectral information available but one may be 3-hydroxyquinine, which is the major metabolite in humans, is pharmacologically active, and is generated by CY3A4 (major) and CYP2C19 (minor).^28–30^ This metabolite is also produced by mouse, rat, and dog, mainly through CYP3A/Cyp3a enzymes.^31^ Finally, we observed a demethylated and glucuronidated metabolite (M3) that was specific to WT mice.

**Figure 7:**
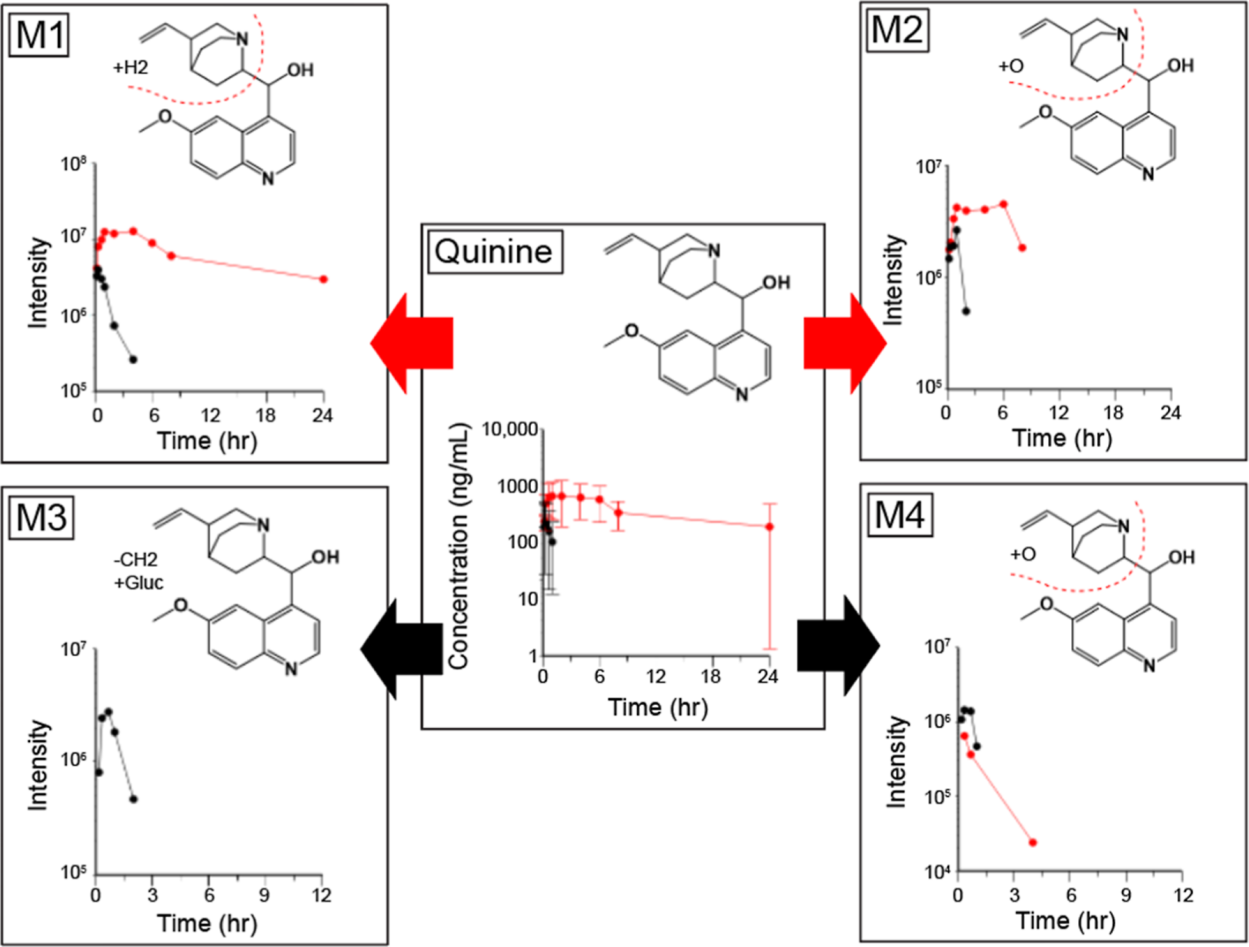
Pharmacokinetics of quinine and metabolites in 8HUM and WT mice. Quinine was administered PO at 20 mg/kg to 8HUM (red) and WT (black) mice (each *n* = 3) and serial blood samples taken for analysis. For parent drug, data shown are mean ± standard deviation of three replicates following quantitative analysis. For metabolites, samples were pooled across time points prior to LCMS data acquisition and values shown are MS peak intensity. Arrows show preferential routes of metabolism for WT (black) and 8HUM (red).

## Discussion

The current critical path between observing in vitro activity against *Plasmodium* in a phenotypic or target-based assay and initiation of clinical trials comprises many steps. In the earliest stages, it may be unknown whether the mode of action (MoA) of the compound(s) under study is sufficient to kill parasites in an infected host, or which of several chemical series under development has the greatest chance of success and should be prioritized above the others. As well as being as an essential step for any compound that may be progressed to preclinical candidate nomination, demonstration of in vivo efficacy for the new target or series is a key early project goal, often termed in vivo PoC. In such a context, the 8HUM *P. berghei* model we have described here constitutes a valuable option available to the researcher for PoC demonstration using compounds that might otherwise have been discarded.

We have previously shown that the 8HUM/Rag2^–/–^ line can be successfully engrafted subcutaneously with human cells, allowing assessment of anticancer drug efficacy.^18^ Here, we investigated whether this model was sufficiently immunocompromised to allow the displacement of murine erythrocytes through intraperitoneal injection of hE, as is commonly used in models such as NSG mice to facilitate efficacy studies against *P. falciparum*. Unfortunately, engraftment was unsuccessful: this did not reach the threshold of 50% hE, which is typically considered appropriate for initiation of compound efficacy study. There are several differences between the immune systems of NSG and 8HUM/Rag2^–/–^ mice. The NOD component of NSG mice is associated with several defects in immunity, while mutation in the Prkdc gene prevents the maturation of T and B cells, and mutation in *Il2rg* prevents cytokine signaling through multiple receptors, ultimately resulting in failure of NK cell maturation. As a combined consequence, the NSG model is one of the most immunodeficient lines currently available. In 8HUM/Rag2^–/–^, deletion of the Rag2 gene also prevents T and B cell maturation, but cytokine and NK cell processes are otherwise undisturbed, providing innate immune response. In an attempt to bring the 8HUM/Rag2^–/–^ immune profile into closer alignment with that of the NSG model, we administered antibodies against the NK cells. Although substantial NK depletion was achieved, we observed no improvement in the engraftment rate, suggesting that, under these conditions, the 8HUM/Rag2^–/–^ line remained relatively immunocompetent. The lower number of NK cells following antibody treatment may nonetheless have been sufficient to mediate rejection. Moreover, cytokine profiling suggested that the circulating levels of key mediators of the immune response were unaffected by NK cell depletion, even after multiple high doses of antibody over a two week period. Hence, we suspended efforts to develop an 8HUM-based *P. falciparum* infection model. In the future, it would be interesting to determine whether genetic deletion of *Il2rg* in 8HUM/Rag2^–/–^ renders this line sufficiently immunocompromised to accept hE engraftment. As this specific modification, with resulting suppression of the interferon response, was a key factor in permitting high rates of engraftment and infection in NSG mice,^11^ any further effort to develop an 8HUM-based *P. falciparum* infection model should prioritize this same genetic deletion ahead of any further attempt to ablate the immune system by alternative means such as antibody blocking, chemical treatment, or surgery.

As an alternative route to implementation of 8HUM within the antimalarial drug discovery workflow, we validated an infection and efficacy experimental format with *P. berghei* in immunocompetent mice. The C57BL/6 strain on which the 8HUM model is based is known to be susceptible to neurological effects associated with cerebral malaria following infection with *P. berghei*;^32^ however, lowering of the initial inoculum and shortening of the experimental time course from the standard approach provided a window sufficient for drug efficacy testing. With initiation of compound administration 24 h after infection followed by monitoring of parasitemia over the subsequent 96 h, this format is very similar to the widely used Peter’s test.^33^ Although not determined here and therefore not validated in the context of 8HUM infection, this model can be used to calculate parameters such as ED_50_, ED_90_, and AUC,ED_90_. Further, a major limitation of this test is the inability to monitor parasite clearance dynamics, i.e., whether a compound acts quickly or slowly. However, we propose that this 8HUM-based variation be given particular consideration for use early in the critical path for antimalarial drug discovery, during the hit-to-lead phase, when PoC in vivo efficacy is required for new chemical series or drug targets to facilitate prioritization and/or progression. In such a context, the researcher should be cognizant of potential differences in aspects such as target structure and MoA between *plasmodium* species, which have developed due to evolutionary divergence. With care and, where possible, mitigation against this risk through selective testing against *P. falciparum* in hE-engrafted mice, the more accessible 8HUM *P. berghei* platform could be used routinely in hit-to-lead and lead optimization phases of development. Further, this model could also be used to refine other in vivo approaches. For example, peripheral blood parasitemia and parasite reduction ratio in mice infected with *P. berghei* have been shown to correlate with human values, allowing prioritization of chemical start-points through relatively brief in vivo screening studies.^22^ By increasing the in vivo exposure of NCEs for which species differences in metabolism are observed, although not necessarily yet known, the use of 8HUM in such a screening approach may increase the proportion of compounds with potential activity in humans that are identified at this early stage for further progression. This new approach therefore constitutes a valuable means to increase the efficiency of antimalarial drug discovery, where resources are often limited.

Previously, we have characterized the in vitro and in vivo metabolism of a wide variety of approved drugs in 8HUM.^13,17^ Here, we extended these investigations to a panel of approved antimalarials. We observed some differences in the in vitro metabolic stability of the artemisinins—artemether, artesunate, and DHA—between human, 8HUM, and WT. For artesunate, CL_int_ was similar between all three experimental groups. Artemether, however, demonstrated differing stability, with CL_int_ in WT > human > 8HUM. As this compound is rapidly metabolized to DHA in humans, and this biotransformation is primarily mediated by CYP3A4,^34^ the difference in turnover between human and 8HUM microsomes may reflect the relative abundance of CYP3A4 in these preparations, with unspecified murine Cyp/s responsible for a more rapid turnover with WT microsomes. Turnover of DHA was similarly low with human and 8HUM microsomes but substantially more rapid with WT microsomes. In the clinic, the main route of metabolic elimination of this drug is through glucuronidation by UGT1A9 and UGT2B7, with CYPs playing no significant role.^35^ Our data may suggest a mouse-specific route of CYP-mediated metabolic elimination of this compound. Due to challenges associated with the bioanalysis of artemisinin derivatives, we did not carry out in vivo studies to define the PK and metabolite profiles of these compounds, but this would be interesting to assess in future studies.

Here, we observed several species differences in the metabolic routes of elimination of quinoline-containing antimalarials. In vitro, quinine, chloroquine, and primaquine were metabolized more rapidly by WT mouse microsomes than by either human or 8HUM microsomes. Metabolite profiling for chloroquine suggested that quinoline oxidation was the primary metabolic basis of this species-specific instability, both in vitro and in vivo. Further, as described in the results section, metabolite profiling suggested that the absence of CYP2C8 in 8HUM and lower expression of CYP3A4 than in human may explain the lower levels of -desethylchloroquine observed in vitro, although this nonetheless appeared to be the major route of metabolic elimination in 8HUM in vivo, as it is in humans.^27^ Previously, we observed that quinidine was less stable with WT mouse microsomes in vitro and that this was likely due to higher rates of O-demethylation.^17^ Here, our in vivo studies with the closely related compound quinine identified a WT mouse-specific combined demethylated and glucuronidated metabolite. Although the structure of this metabolite could not be determined, it may represent a subsequent phase 2 event on the same metabolic pathway. We observed much higher levels of primaquine in 8HUM than in WT mice. In humans, carboxy-primaquine is produced by monoamine oxidase,^36^ and this is the major metabolite found in plasma.^37^ However, primaquine is effectively a prodrug in humans, as hydroxylation by CYP2D6 generates 5-hydroxyprimaquine and 5-hydroxy-6-desmethylprimaquine, active metabolites that are highly potent against both liver and sexual transmission stages of *P. falciparum*.^38,39^ Here, metabolites conforming to carboxy-primaquine and hydroxy-primaquine were more abundant in 8HUM mice than in WT mice. CYP2D6 is expressed at a higher level in 8HUM than in humans, with high activity,^40^ and it is tempting to speculate that this metabolite may therefore be 5-hydroxyprimaquine, but further investigation with authentic standards would be required to confirm this and the high level of this metabolite in 8HUM may be due, at least in part, to the relatively high levels of parent drug in circulation. As described in the results section, the high stability of amodiaquine in 8HUM microsomes as compared to that in human and WT mouse microsomes could potentially be explained by the different enzymes present in each system. In humans, this compound is rapidly N-desethylated by CYP2C8,^23,24^ an enzyme absent from 8HUM. Further, the difference between 8HUM and WT mice could be due to the activity of murine Cyps absent from the former. In summary, as might be expected given the diversity of metabolic routes of drug elimination across species, the use of 8HUM does not improve the clinical alignment for every compound. In some cases, alignment may in fact be poorer than when using WT mice, as was seen with amodiaquine. Overall, however, these results demonstrate that the metabolism of antimalarials in 8HUM is more clinically relevant than that in WT mice and that this applies to both the rate of metabolic elimination and the profile of metabolites generated. As previously described,^17^ implementation of 8HUM should involve an in vitro microsomal metabolic stability assay as a first step, which will rapidly identify any compounds for which no advantage is conferred.

## Conclusion

Here, we demonstrate how the 8HUM model can be used during antimalarial drug discovery to bypass species differences in drug metabolism. This will increase the efficiency of the earliest stages of development, where molecules that show activity against *Plasmodium* in vitro must subsequently demonstrate in vivo activity in a mouse model of infection. Utilization of the short-course 8HUM *P. berghei* infection model described herein to demonstrate such efficacy prior to extensive compound optimization will expedite prioritization of the most promising drug targets and chemical series. In addition, we have shown that the use of 8HUM improves the alignment of the metabolism of approved antimalarials with the clinical situation, suggesting that translational modeling of efficacy and toxicity will be improved for combinations including these compounds, as well as for NCEs. Integration of 8HUM into existing workflows will therefore increase the chances of success in antimalarial drug discovery programs.

## Materials and Methods

### Chemicals and Reagents

All compounds were purchased from Sigma-Aldrich/Merck (Burlington, MA, USA), with the exception of pyrimethamine, which was kindly provided by Delphine Baud at Medicines for Malaria Venture. For in vitro studies, compounds were solubilized in dimethyl sulfoxide (DMSO) to 10 mM and stored under nitrogen at ambient temperature in the Compound Management facility at the University of Dundee Drug Discovery Unit. Aliquots were used within 3 months of solubilization. Human hepatic microsomes (mixed gender pool from 150 individuals) were purchased from Gibco (Thermo Fisher Scientific). All LC–MS/MS mobile phase reagents were purchased from Fisher Scientific (Thermo Fisher Scientific).

### Animal Ethics and Husbandry

The 8HUM and 8HUM/Rag2^–/–^ breeding colonies were maintained at Charles River Laboratories (Margate, UK). Mice were housed in fully flexible isolators with Aspen flake bedding and a conditioned and HEPA-filtered air supply, a 13 h light/11 h dark cycle, and ad libitum access to water (filtered and chlorinated with sodium hypochlorite) and food (FRDB-pelleted diet). Temperature and relative humidity were maintained at 21 ± 1 °C and 55% ± 10%, respectively. All consumables were irradiated at a minimum of 35 kGy or sterilized by an autoclave and/or ethylene dioxide prior to use. [Note on the C57 WT origin.]

All animal experiments carried out at GSK were performed at the AAALAC-accredited Laboratory Animal Science facility in Tres Cantos (Madrid, Spain). All experiments were approved by the GlaxoSmithKline Global Health Research and Development Medicines Group Ethical Committee. All animal studies were ethically reviewed and carried out in accordance with the European Directive 2010/63/EU and the GSK Policy on the Care, Welfare, and Treatment of Animals. Experimental and control animals infected with *P. berghei* were euthanized at the end of the assay (day 5 after infection), before developing severe malaria, and all efforts were made to minimize suffering. Experimental and control animals were specific pathogen-free 8–12 week old females, body weight range 20–22 g. CD1 Swiss (Hsd:ICR) mice were obtained from Harlan Interfauna (Iberica, Spain). Immunodeficient NSG (NOD.Cg-Prkdcscid Il2rgtm1Wjl/Sz) mice were obtained from Charles River Laboratories (L’Arbresle, France, under license of The Jackson Laboratory, Bar Harbor, Maine, USA). Up to five animals were accommodated in Tecniplast type IV cages with autoclaved dust-free corncob bedding (Panlab, Barcelona, Spain). Facilities were kept under a 12 h light/dark period at a room temperature of 22 ± 2 °C and 40–70% relative humidity and air-conditioned with 20 air changes per hour. Filtered tap water and a γ-irradiated pelleted diet were provided ad libitum.

All animal experiments carried out at the University of Dundee were approved by the Welfare and Ethical Use of Animals Committee (approval No. WEC2019_008) and carried out under the Animals (Scientific Procedures Act) 1986, as amended in 2012, and in accordance with the European Union Directive 2010/63/EU. Animals were inspected regularly by staff trained and experienced in small animal husbandry, with 24 h access to veterinary advice. Mice were maintained in filter top cages (Thoren Mouse Caging System, Thoren) containing Eco-Pure chip2HK (Datesand Group) for bedding with ad libitum access to food (RM1; Special Diet Services) and water and a 12 h light/dark period. Temperature and relative humidity were maintained between 20 and 24 °C and 45% and 65%, respectively. Experimental design was guided by power calculations (G*Power; https://www.gpower.hhu.de), pilot experiments, and previous experience and was undertaken in line with the 3Rs principles of replacement, reduction, and refinement (https://www.nc3rs.org.uk).

### *P. falciparum* Infection and Efficacy

Human biological samples were sourced ethically, and their research use was in accord with the terms of informed consent under an IRB/REC-approved protocol. Erythrocyte concentrates from malaria-negative donors were provided by Biobanco del Centro de Hemoterapia y Hemodonacion de CyL (Castilla y Leon, Spain) and CTCM (Centro de Transfusiones de la Comunidad de Madrid, Spain). Before injection, erythrocyte concentrates were washed twice with washing buffer (RPMI 1640 (Sigma) containing 25 mM Hepes (Sigma) and 3.1 mM hypoxanthine (Sigma)) at 700*g* for 10 min at ambient temperature. Buffy coat was removed by aspiration, and erythrocytes were resuspended at 50% hematocrit (25% washing buffer and 25% inactivated human AB serum (Sigma)). The blood suspension was warmed at 37 °CC for 20 min before intraperitoneal injection with 1 mL of hE suspension every day during the experiments. During initial evaluation, groups of 8HUM/Rag2^–/–^ and NSG (each *n* = 25) were injected with hE daily for 19 days. In those mice taken forward for infection, 2 × 10^7^ hE infected with *P. falciparum* 3D7^0087/ND9^ were administered intravenously in a final volume of 0.3 mL in sterile saline solution (0.9%) prewarmed to 37 °C.

To evaluate parasitemia, 2 μL of peripheral blood was added to 94 μL of saline solution (0.9%) containing 5 μL of SYTO-16 (Invitrogen, S-7578) and 1 μL of mAbter119-PE (eBioscience, 12-5921-81) in 96-well V-bottomed plates. Following incubation for 20 min at ambient temperature, samples were fixed with the addition of 10 μL of glutaraldehyde (0.25% v/v) per well and analyzed by flow cytometry using a FACS Calibur flow cytometer (Becton Dickinson, San Jose, CA) with CellQuest Pro (version 5.1). During acquisition, the percentage of infected hE was determined as those events that were positive for SYTO-16 and negative for mAb TER119-PE, relative to the total cell region previously selected in a dot plot of 180° dispersed light (forward scatter-FSC) opposite to 90° dispersed light (side scatter-SSC). The percentage of infected erythrocytes was calculated as the SYTO-16 positive/TER119-PE negative events in an FL1/FL2 dot plot. Erythrocytes and leukocytes were gated in logarithmic forward and side dot plots. Green and red fluorescence were detected in the corresponding FL-1 and FL-2 photomultipliers through a 530/30 or 585/42 band-pass filter, respectively. The mean fluorescence channels in FL-1 and FL-2 were adjusted to equal values in the first decade of intensity in bivariate logarithmic scale dot plots with noninfected erythrocytes. Leukocytes could be identified and excluded during analysis in standard saline citrate/FL-1 dot plots due to their higher level of nucleic acids than infected erythrocytes. Compensation of SYTO-16 emission in FL-2 and TER119-PE in FL-1 was achieved to accurately set up the region of infected events. This region must be defined by comparison of blood samples from uninfected-engrafted and infected-engrafted IL2 mice by increasing the compensation of SYTO-16 emission in FL-2 until obtaining a defined region for infected events. Data analysis was performed by using CellQuest Pro software.

Cytokines were quantified using CBA Th1, Th2, and Th17 and inflammatory kits (BD) according to manufacturer’s instructions. Briefly, 50 μL of serum was mixed with 50 μL of a capture bead mixture containing beads for each cytokine to be detected, and 50 μL of the mouse inflammation PE detection reagent was added. Samples were incubated for 2 h in the dark at ambient temperature. For quantification, a standard curve was prepared by serial 3-fold dilutions of the cytokine standards. Samples were analyzed using a Fortessa X-20 flow cytometer (BD) at medium speed and at least 8000 events per stopping gate. Cytokines were detected in an FL2/FL3 dot plot. The intensity of fluorescence in the FL2 detector indicated the quantity of each cytokine present in the sample, while the FL3 detector identified each cytokine depending on the fluorescence intensity of the capture beads provided in the kit. Once acquired, samples were analyzed using FCAP Array and FlowJo Software (BD).

### Antibody Treatment and Flow Cytometry

Antimouse NK1.1 (InVivomAb, BioXcell BE0036) or IgG2a isotype control (InVivomAb, BioXcell BE0085) was prepared to a final volume of 200 μL/dose in an InVivoPure pH 7.0 Dilution Buffer (BioXcell) and administered i.p. at either 200 or 600 μg/dose as described. During the engraftment study, 8HUM/Rag2^–/–^ was administered anti-NK1.1 on days −3, −1, 1, 4, 7, 10, 14, and 17, relative to the first hE injection on day 0. Engraftment with hE was continued with once daily injections for 19 days. Blood samples for analysis were collected on the same days as antibody administration and additionally on days 21 and 25. Whole blood was obtained postmortem by cardiac puncture with a heparinized syringe and collected into heparinized tubes. Following dilution with phosphate-buffered saline (PBS), cells were added to a 15 mL tube containing Ficoll (Ficoll–Paque Premium, Cytiva 17-5442-02) such that the final ratios of blood, PBS, and Ficoll were 1:1:1. Samples were centrifuged at 500*g* for 30 min at 21 °C, and the interphase, containing immune cells, was transferred to a fresh 15 mL tube. Cells were washed twice with PBS and resuspended in 1 mL of medium (RPMI-1640 containing l-glutamine and 10% heat-inactivated FBS) for counting using a CASY Cell Counter & Analyzer (OMNI Life Science). Spleens were collected into a 6-well plate. Following homogenization through a 40 μm cell strainer into 10 mL of medium, cells were pelleted by centrifugation at 300*g* for 5 min at 4 °C. Erythrocytes were lysed by adding 4 drops of tap water to the pellet and mixing gently for approximately 15 s, followed by addition of medium to a 15 mL total volume and centrifugation at 300*g* for 5 min. Pellets were resuspended in 1 mL of medium for cell counting.

Labeling for flow cytometry was carried out at 4 °C and protected from light using aliquots of 50,000 cells with 100 μL of labeling solution (PBS with 0.5% FBS) containing antibodies at the following concentrations: CD45 (Biolegend 157,620) at 0.6 μg/1 × 10 cells and NKp46/NCR1 (BioTechne FAB22252P) at 5 μg/1 × 10 cells. Labeled cells were collected by centrifugation and fixed by resuspension in 200 μL of 4% paraformaldehyde (PFA) in PBS for 15 min at ambient temperature, protected from light. Fixed cells were washed twice with PBS containing FBS and resuspended in 300 μL of PBS–FBS for analysis. Samples were analyzed using a FACS Symphony A1 cytometer (BD) and FACSDiva software (v9.0.2). During acquisition, leukocytes were considered those events included in the BV421-A positive region related to the total cell region previously selected in a dot plot in a linear scale of 180° dispersed light (forward-FSC) opposite to 90° dispersed light (side-SSC). The NK cells were considered those events double positive for CD45-Pacific Blue (BV421 detector) and NKp46/NCR1-PE (FL2 detector) in an FL2/BV421 dot plot. Unstained cells and cells stained with each of the antibodies used alone were used to determine the acquisition parameters and compensation values.

### *P. berghei* Infection and Efficacy

Donor mice (CD-1) were intraperitoneally administered a solution containing cryopreserved erythrocytes infected with *P. berghei* ANKA (200 μL at approximately 4% parasitemia). After 3 days, erythrocytes were harvested by cardiac puncture for intravenous administration to 8HUM mice at 0.3–1.5 × 10^6^ cells/mouse. To evaluate parasitemia in 8HUM at the indicated times, 2 μL of peripheral blood was added to 95 μL of saline solution (0.9%) + 5 μL of SYTO-16 (Invitrogen, S-7578) in 96-well V-bottomed plates. Following incubation for 20 min at ambient temperature, samples were fixed with the addition of 10 μL of glutaraldehyde (0.25% v/v) per well and analyzed by flow cytometry using a FACS Calibur flow cytometer (Becton Dickinson, San Jose, CA) with CellQuest Pro (v5.1). Piperaquine (Sigma, C7874) was administered at 7 mg/kg in aqueous vehicle containing 1% (w/v) methylcellulose and 0.5% (w/v) Tween 80. Peripheral blood samples were collected daily, and parasitemia was determined as described.

### Microsome Incubations for Stability and Metabolite Profiling

Microsomes from 8HUM were prepared as described previously.^17^ Compounds were added at 0.5 μM (metabolic stability) or 5 μM (metabolite profiling) to microsomes in buffer (0.5 mg/mL protein in 50 mM potassium phosphate, pH 7.4), and reactions were initiated with the addition of excess NADPH (final concentration 0.8 mg/mL). Aliquots (50 μL) were taken immediately and at 3, 6, 9, 15, and 30 min, mixed with two volumes of acetonitrile containing internal standard (donepezil, 50 ng/mL), and kept on ice. After all samples were collected, 250 μL of 20% acetonitrile was added to each sample and samples were then centrifuged for 10 min at 3000*g* at ambient temperature. LC–MS/MS analysis was carried out immediately.

### Pharmacokinetic Experiments

Three female mice were used in each pharmacokinetic experiment. Dose levels and schedules were as described in the text. All compounds were administered as fine suspensions in a vehicle of 1% methylcellulose and 0.5% Tween80 in water at a dose volume of 5 mL/kg. Serial blood samples (10 μL) were taken from the tail vein at the time points shown, diluted into 9 volumes of Milli-Q water, and stored at −20 °C prior to bioanalysis. PK parameters were determined by noncompartmental analysis using the Phoenix WinNonlin version 8.3.1.5014 (Certara).

### LC–MS/MS Analysis of Samples

Microsomal incubation samples were analyzed on an Acquity UPLC system coupled to a Xevo TQS-Micro, operated using MassLynx software version 4.2 (Waters, Wilmslow, UK). Chromatographic separation was achieved using an Acquity UPLC BEH C18 column, 50 × 2.1 mm, a particle size of 1.7 μm (Waters, part number 186002350) held at 45 °C, with mobile phase A of 0.01% formic acid in Milli-Q water, and mobile phase B of 0.01% formic acid in LC–MS grade methanol. The gradient program was as follows: 0.0–0.3 min: 5% B, 0.3–1.3 min: 5–95% B, 1.3–1.8 min: 95% B and 1.8–1.81 min: 95–5% B. Samples from PK studies were analyzed on an Acquity UPLC system coupled to a Xevo TQ-XS, using the same column as above (Waters). The column was held at 40 °C, mobile phase A was 0.01% formic acid in LC–MS grade water, mobile phase B was 0.01% formic acid in LC–MS grade acetonitrile, and the gradient program was as follows: 0.0–0.5 min: 5% B, 0.5–2.0 min: 5–95% B, 2.0–2.5 min: 95% B and 2.5–3.4 min: 95–5% B. The flow rate was 0.6 mL/min for all methods. The mass spectrometer was operated with electrospray ionization in positive mode, with a capillary voltage of 0.8 kV, a desolvation temperature of 500 °C (in vitro samples) or 600 °C (PK samples), a desolvation gas flow at 1000 L/h, a cone gas flow at 150 L/h, and a source temperature at 150 °C. Multiple reaction monitoring transitions, cone voltages, and collision energies were optimized for each compound using QuanOptimise software.

### Intrinsic Clearance Determination

Raw LC–MS/MS data were exported to XLfit (IDBS, Woking, UK) for calculation of the exponential decay rate constant (*k*) from the ratio of the peak area of the test compound to the internal standard at each time point. Intrinsic clearance (CL_int_) was calculated by multiplying k by the incubation volume and the microsomal protein concentration. Verapamil was used as a positive control to confirm acceptable assay performance, and all compounds were run in singlicate. Lower and upper limits of quantitation were 0.011 and 0.1 mL/min/mg, respectively. Values were transferred to RStudio (Posit, Boston, MA, USA) for visualization using the ggplot2 package.^41^

### Quantitative Bioanalysis of PK Samples

On the day of sample analysis, calibration standards (CSs) and quality controls (QCs) were prepared from separate compound aliquots. Compounds were dissolved in DMSO and spiked into diluted blank blood as a control matrix, prior to extraction with three volumes of acetonitrile containing the internal standard (donepezil at 5 ng/mL), in parallel with PK samples. Concentration of the drug in samples was determined by interpolation onto CS values using TargetLynx (Waters). Acceptance criteria of the bioanalytical method included a CS accuracy of ±20% of nominal (theoretical) concentration across the range of interest. Lower limit of quantification was determined as the analyte response with ≥ three times the analyte response of single blanks (extracted blood containing IS but no test compound). QCs at low-, medium-, and high- concentration levels were injected throughout the analytical run, with an acceptance criterion of 66% of injected samples being within ±20% of nominal concentrations. All single and double blanks (extracted blood with no IS or test compound) were verified as free of interference at the retention times of the IS and test compound. Sample carryover was determined in a blank injection immediately following the injection of the top CS. Carryover was deemed accepted when the test compound response in the blank was <1% of that in the top calibration sample.

### Metabolite Profiling

Samples were analyzed using a Vanquish UHPLC system interfaced with an Exploris 120 mass spectrometer, operated using Xcalibur version 4.4.16.14 (Thermo Fisher Scientific, Waltham, MA, USA), as described previously.^17^ Briefly, chromatography was carried out using a Hypersil Gold C18 column, 50 × 0.21 mm, 1.9 μM particle size (Thermo Scientific, part number 25002-052,130), held at 40 °C, with a mobile phase of 0.01% formic acid in LC–MS grade water (A) and 0.01% formic acid in LC–MS grade acetonitrile (B). Flow rate: 0.5 mL/min. The mass spectrometer was operated with electrospray ionization in positive mode with a full scan resolution of 30,000, a scan range of 120–1200 *m*/*z*, and top four ion selection for data-dependent MS2 acquisition. Acquired data were processed in Compound Discoverer version 3.2 (Thermo Fisher Scientific). Putative metabolites were annotated based on mass accuracy against nominal (±3 ppm tolerance), isotope pattern, and feasibility of biotransformation, with additional monitoring of peak shape and MS intensity relative to sampling time. Structural elucidation was carried out through interpretation of fragmentation spectra and in consideration of known metabolites of the compounds under study, as described in FDA and EMA review documentation, and published studies. Data were transferred to RStudio or Phoenix WinNonlin for visualization.
